# Signatures of chaos in animal search patterns

**DOI:** 10.1038/srep23492

**Published:** 2016-03-29

**Authors:** Andy M Reynolds, Frederic Bartumeus, Andrea Kölzsch, Johan van de Koppel

**Affiliations:** 1 Rothamsted Research, Harpenden, Hertfordshire, AL5 2JQ, UK; 2 Theoretical and Computational Ecology Lab (CEAB-CSIC). Blanes 17300, Spain; 3 CREAF, Cerdanyola del Vallès, 08193 Barcelona, Spain; 4 Department of Migration and Immuno-Ecology, Max Planck Institute for Ornithology, Am Obstberg 1, 78315 Radolfzell, Germany; 5 Department of Biology, University of Konstanz, Universitätsstraße 10, 78464 Konstanz, Germany; 6 Department of Estuarine and Delta Systems, NIOZ Royal Netherlands Institute for Sea Research and Utrecht University, P.O. Box 140, 4400 CA Yerseke, the Netherlands; 7 Institució Catalana de Recerca i Estudis Avançats (ICREA), 08010 Barcelona, Spain; 8 Department of Animal Ecology, Netherlands Institute of Ecology (NIOO-KNAW), P.O. Box 50, 6700 AB Wageningen, The Netherlands; 9 Groningen Institute for Evolutionary Life Sciences (GELIFES), University of Groningen, P.O. Box 11103, 9700 CC Groningen, the Netherlands

## Abstract

One key objective of the emerging discipline of movement ecology is to link animal movement patterns to underlying biological processes, including those operating at the neurobiological level. Nonetheless, little is known about the physiological basis of animal movement patterns, and the underlying search behaviour. Here we demonstrate the hallmarks of chaotic dynamics in the movement patterns of mud snails (*Hydrobia ulvae*) moving in controlled experimental conditions, observed in the temporal dynamics of turning behaviour. Chaotic temporal dynamics are known to occur in pacemaker neurons in molluscs, but there have been no studies reporting on whether chaotic properties are manifest in the movement patterns of molluscs. Our results suggest that complex search patterns, like the Lévy walks made by mud snails, can have their mechanistic origins in chaotic neuronal processes. This possibility calls for new research on the coupling between neurobiology and motor properties.

It has long been recognized that the key to understanding movement patterns lies with elucidating the underlying generative processes^1^. An important step to be made here is to distinguish the intrinsic movement behaviour of the animals from the observed movement patterns which are often confounded by a multitude of responses to environmental cues^2^,^3^. Lévy walks (LW) have been proposed as intrinsic, optimized movement patterns for searching for food or other search targets that are either rare or hard to observe^4^,^5^. Signatures of LWs have been observed in a diverse range of organisms, including bacteria, T cells, mussels, mud snails, honeybees, sharks, turtles, jellyfish and other marine predators, wandering albatrosses, extinct marine organisms that once occupied ancient sea beds and even in human hunter-gatherers^6^,^7^,^8^,^9^,^10^,^11^,^12^,^13^,^14^,^15^,^16^. LW alternate clusters of many short steps with longer steps between them, creating fractal movement patterns that have no characteristic scale. The self-similar, fractal properties of LW can be advantageous when randomly searching, and as a result there could be natural selection for LW^4^,^5^. Nonetheless, the concept of Lévy search behaviour has been met with fierce resistance^17^ in large part because of the absence of underlying neurological or physiological processes that can explain the emergence of this complex, scale-free behaviour.

Studies on invertebrates have in the past years provided experimental evidence for LW movement patterns in ants (*Melophorus bagoti*), mussels (*Mytilus edulis*) and mud snails (*Hydrobia ulvae*), again highlighting that the simple neurological processes in lower animals can provide an interesting window on mechanisms that create scale-free movement patterns^6^,^12^,^18^. However, understanding the biological basis for motor control requires a coupling between animal movement patterns and neurological/physiological processes. Such couplings are largely absent from the literature; although some attempts have been made to link the spontaneous flight patterns of *Drosophila* fruit flies to spontaneous neuronal-firing activity in the central complex^19^. There is, for instance, a profusion of literature on the neurobiology of molluscs^20^,^21^,^22^ reporting on chaotic dynamics, but there are no studies which have tested for these properties in their movement patterns which can have LW characteristics^6^,^12^, i.e., tested whether the timing of turns in movement patterns are chaotic. Cole^23^ did, however, find signatures of chaos in the activity patterns of *Lepthothorax allardycei* ants and these were subsequently attributed to neuronal processes determining when to search, rather than how to search^24^. Hayashi *et al*.^20^ provided the first evidence for neurons having chaotic dynamics. They uncovered chaos in recordings of the electrical activity of the giant silent neuron in the esophageal ganglion of the mollusk *Onchidium verruculatum*. Their evidence took the form of a ‘strange attractor’ which they reconstructed from the data. Strange attractors are data-derived mathematical constructs that are indicative of chaotic dynamics. Komendantov and Kononenko^22^ later uncovered signatures of chaos in a mathematical model of pacemaker activity in the bursting neurons of a snail, *Helix pomatia*.

Here we show that chaotic temporal dynamics of the kind evident in the neurons of molluscs^20^,^21^,^22^ can be discerned in the movement behaviour of mud snails while searching under controlled conditions. Moreover, we show that these movement patterns can be approximated by Weierstrassian random walks (also known as Weierstrassian Lévy walks). Weierstrassian random walks comprise a hierarchy of nested movement patterns which if extended indefinitely would correspond to a LW. Weierstrassian random walks are one of the simplest random walks which do not satisfy the central limit theorem and have come to epitomize scale-invariance^25^. Although initially regarded as a mathematical abstraction^26^, Weierstrassian random walks have been linked mathematically to dynamical chaos^27^,^28^. The results presented in this paper specifically relate Lévy search behaviour of mud snails to chaotic dynamics and suggest that the emergence of Lévy walk type of patterns can, in fact, be attributed to neurobiological chaos. The advance leaves open for further research identification of the neuronal generative mechanism.

## Methods

### Classification of movement patterns

We used a dataset of 36 individual recordings of mud snails moving in featureless experimental arenas^12^. The snails were starved for 1 h, marked with a yellow spot for track recognition and then placed in an arena of bare mud, representing their natural foraging habitat. Movements paths were recorded using a webcam (Logitech QuickCam 9000 Pro) which was set to take pictures every 10 s for 5 h. These pictures were then loaded into a bespoke Matlab programme that automatically followed the yellow spot and recorded its positions. This process was supervised by a student and if necessary adapted by hand. Previously, Kölzsch *et al*.^12^ demonstrated the presence of Lévy patterns in the search movement of these mud snails, but the authors did not test for the presence of Weierstrassian random walks. For each individual, we aggregated regularly sampled snail tracks into sequences of ‘steps’, i.e., into quasi-linear track segments during which the snail did not change direction significantly. This was done using the approach of Humphries *et al*.^29^ in which the movement patterns are first projected onto the *x*- and *y*-axes to create two one-dimensional movement patterns for each individual. Turns in these projections can then be identified in an unambiguous way as occurring where the direction of travel changes. Without projection, turns can only be identified by making reference to arbitrarily defined critical-turning angles^29^. Humphries *et al*.^29^ showed that projection does not affect the functional form of the step-length distribution. Resulting step-length distributions were fitted to truncated power-laws, truncated exponentials, and multi-exponentials using maximum likelihood methods^30^,^31^. These results were robust with respect to other definitions of turning angle^12^. Power-laws are indicative of true LW, exponentials are null models of the movement patterns, and the multi-exponentials are indicative of multi-modal walks and also Weierstrassian random walks when model parameters satisfy certain scaling-relations^32^.

### Hallmarks of chaos

It is now generally accepted that a unique intrinsic and observable signature of systems exhibiting deterministic chaos^33^ is a fluctuation power spectrum with an exponential frequency dependency^34^,^35^,^36^. Such exponential decays are commonly observed in theoretical models of chaos and in experimental studies of fluid flows and confined plasmas^37^,^38^,^39^,^40^. These spectrum have been traced to the presence of pulses (bursts of activity) occurring in the intermittent time dynamics having a characteristic ‘Lorentzian’ form 
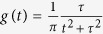
 where *t* is time and where the constant *τ* determines the magnitude and the width of the pulse^41^,^42^. Time-series containing only pulses of width *τ* are characterized by a simple exponential power spectrum 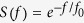 where the characteristic frequency *f*_0_ = 1/*πτ*. Stretched exponentials can arise when pulse widths are broadly distributed^43^,^44^. By way of contrast white noise processes (i.e. Poisson processes and all other processes which have no temporally correlated behaviour) have flat spectrum whilst ‘1/f’ noise (found in scale-invariant systems with long-range correlations) have spectrum with power-law frequency dependency^45^.

### Testing for the presence of chaos

We looked for these hallmarks of chaos in the time-series data of the tracked mud snails. The extracted 1-dimensional turns define time-series *u*(*t*). If, for example, turns occurred at times *t* = 3Δ*t*, 5Δ*t*, 6Δ*t*, …, (*N* − 2)Δ*t*, *N*Δ*t* where Δ*t* is the time interval between consecutive positional fixes and *N* is the number of positional fixes then the entries in the time-series *u*(*t*) would be 0, 0, 1, 0, 1, 1, …, 1, 0, 1. The power-spectrum of *u*(*t*) is the square of the magnitude of the Fourier transform of *u*(*t*) and is given by


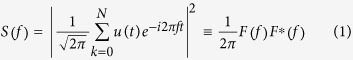


where *t* = *k*Δ*t* is the time at which the k^th^ positional fix was made; *f* is frequency, *F*( *f*  ) is the discrete Fourier transform of *u*(*t*), and F* ( *f* ) is its complex conjugate. Spectra were fitted to stretched exponentials, 
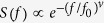
 using maximum likelihood methods. Good fits to stretched exponentials would be indicative of the presence of chaos, poor fits would be indicative of the absence of chaos.

To test for the presence of Lorentzian pulses, tracks were partitioned into clusters of small movement patterns (called ‘search clusters’ in Kölzsch *et al*.^12^ and indicative of intrinsic area-restricted searching behaviours) and long movements. That is, after each turn in the movement pattern, the number of subsequent turns were counted, and the normalized cumulative numbers of turns across time, 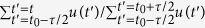, were compared with the expectations for a Lorentzian pulse of width *τ* centred on time *t*_0_:


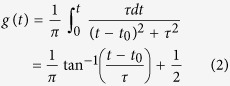


Pulses (i.e., cumulative frequency distributions) were identified using the Kolmogorov-Smirnov test, and grouped together according to their width.

We further tested for chaos by calculating the largest ‘Lyapunov exponents’. These exponents characterise the separation of initially close *state-space* trajectories. Here they were calculated using the method of Rosenstein *et al*.^46^ which we now outline, as it was fully documented by Rosenstein *et al*.^46^ In this approach the state-space trajectories are expressed as a matrix *U* = [*U*_1_, *U*_2_, …, *U*_3_]^*T*^ where *U*_*i*_ = [*u*_*i*_, *u*_*i*+1_, …, *u*_*i*+(*m*−1)_], *i* denotes the time-step and *m* = 10, known formally as the ‘embedding dimension’ is effective of the degree of autoregression in the analysis. The algorithm locates the *nearest neighbour* of each point on the trajectory. The nearest neighbour of *U*_*j*_ is found by searching for a point, 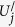, that minimizes the distance to *U*_*j*_. This initial distance from *U*_*j*_ can be expressed as 
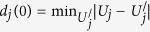
. The largest Lyapunov exponent is then estimated from how the distance, *d*_*j*_(*i*), between the pair of nearest neighbour points, *U*_*j*_ and 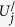, grows with time *t* = *i*Δ*t*. The presence of chaos is indicated by a separation that increases exponentially in time as *d*_*j*_(*i*) ∝ *e*^*λ*(*i*Δ*t*)^ where *λ* is the largest Lyapunov exponent. In practice the presence of chaos is indicated by linear growth of 〈log *d*_*j*_(*i*)〉 where 〈…〉 denotes the average over all values of *j*. The averaging is crucial when using small, noisy data sets. Note also that the growth of 〈log *d*_*j*_(*i*)〉 is expected to saturate at long times since the system is bounded in phase space and the average divergence cannot exceed the “length” of the attractor^46^.

## Results

### Classification of movement patterns

**Figure 1:**
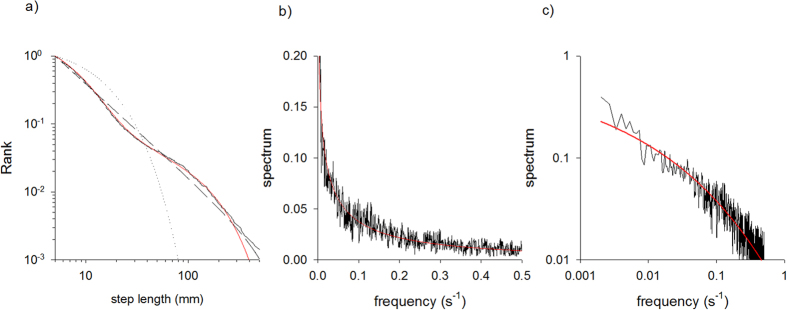
(**a**) Rank frequency distribution of step lengths in the movement pattern data (black solid-line) together with the best-fit exponential (dotted-line), power-law (dashed) and tri-exponential (red solid-line). Data are pooled for all 36 recorded individual movement patterns moving within a featureless arena. (**b**) Spectrum (black solid-line) together with the best-fit stretched exponential (red solid-line) on linear-linear scales and (**c**) on log-log scales.

Consistent with the analyses of Kölzsch *et al*.^12^ the step-length distribution is found to be best modelled as a tri-exponential (Fig. 1a). The mean step-lengths, 〈*l*_1_〉 = 0.208, 〈*l*_2_〉 = 0.043 and 〈*l*_3_〉 = 0.009, in the three exponentials do, in fact, satisfy a simple scaling relation: 〈*l*_2_〉 = 〈*l*_1_〉^2^ and 〈*l*_3_〉 = 〈*l*_1_〉^3^. This scaling is the hallmark of a truncated Weierstrassian random walk^32^. The weightings of the exponentials are 0.88, 0.08 and 0.04. As a consequence our fitted Weierstrassian random walk corresponds to a truncated Lévy walk with Lévy exponent *μ* = 2.5^32^. The tri-exponential fit obtained by Kölzsch *et al*.^12^ also resembled a power-law (and so is indicative of Lévy type of walking) but it is not a Weierstrassian Lévy walk and does not fit the observational data as well, i.e., it has a smaller log-likelihood. This is due to the fact that likelihood landscapes may show several local minima. Using much more computational power to explore such a likelihood landscape we found a better fit that matches a Weierstrassian Lévy walk. The log-likelihood values for the two fittings are quite close to one another with values −16882.2 and −16901.0 respectively. We found no support for multi-exponentials with 4 terms.

### Identification of chaos

**Figure 2:**
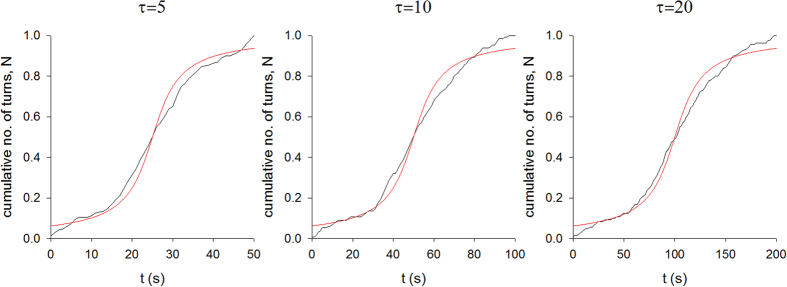
Averaged normalized cumulative counts of turns in 3 differently-sized ‘search clusters’ (black solid-lines) together with the cumulative number of turns in same-sized Lorentzian pulses (red solid-lines) (Eqn. 2).

**Figure 3:**
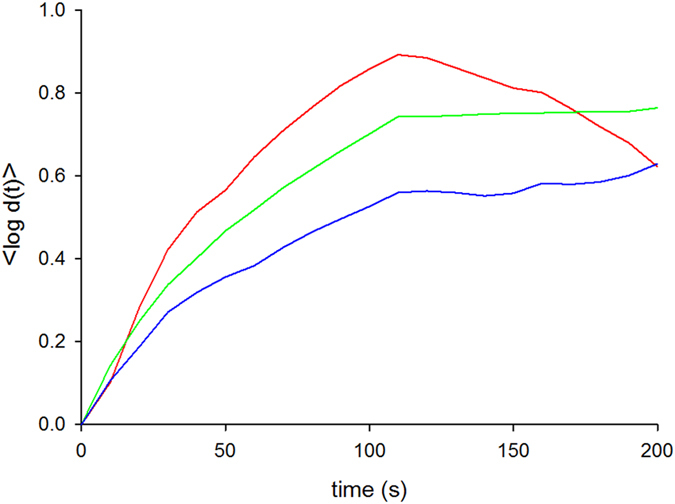
Examples of the average log separation of initially close *state-space* trajectories shows exponential divergence. This divergence is indicative of the presence of a positive Lyapunov exponent and so the presence of deterministic chaos (see text). Different colours indicate tracks of different individuals.

The power spectrum of the mud snails’ turn sequences is very well represented by a stretched exponential with *ν* = 1/4 (Fig. 1b,c). The occurrence of such a power spectrum can be attributed to the presence of Lorentzian pulses, since the number of turns within search clusters are well represented by the theoretical expectations, Eqn. 2, for such pulses (Fig. 2). These two characteristics are the hallmarks of deterministic chaos. Furthermore, we conclude positivity of the Lyapunov exponents from exponential divergence of initially close state-space trajectories, which provides further support for the presence of deterministic chaos (Fig. 3).

## Discussion

There is now compelling evidence that many organisms have movement patterns which can be approximated by LW^6^,^7^,^8^,^9^,^10^,^11^,^12^,^13^,^14^,^15^,^16^. There is, however, little understanding of the processes that underlie these LW, although many putative, biologically plausible mechanisms have been suggested^47^. Using an invertebrate species with limited perceptive abilities, mud snails, we have revealed clear signatures of chaotic dynamics within their long-tailed movement patterns made under controlled experimental conditions. We hypothesize that the movement of this invertebrate is driven by chaotic neurological activity, creating fractal movement patterns that optimize search success. Our work thereby provides an exciting new perspective on both the neurological and evolutionary origins of LW and their prevalence amongst invertebrate species.

The ubiquitous nature of deterministic chaos in nervous systems suggests that inherent multi-scale movement patterns, resembling LW, could be more common than current observations indicate. Power-law distributions of spontaneous neuron firing signals have been observed widely in *in vitro* studies^48^,^49^,^50^. Such spontaneous electrical activity could generally provide the timing signals necessary for the execution of multi-scale displacements and may account for LW-like movement patterns in *Drosophila* fruit flies^19^,^51^. In fruit flies, locomotor activity is coordinated by a region called the central complex, casually referred to as the insect’s “motor cortex”. Martin *et al*.^19^ found that blocking synapses within the ellipsoid-body, a sub-region of the central complex, leads to a loss of the fractal (i.e., Lévy-like) properties of adult walking behavior. Maye *et al*.^51^ subsequently reported on fractal order (resembling Lévy flights) in the temporal structure of spontaneous flight manoeuvres in tethered *Drosophila* fruit flies, prompting them to suggest that these organisms are both deterministic and chaotic. These traits were later found in moths and bees which suggests that they are common amongst invertebrates^3^.

### Chaos in Lévy walks

LW, named after the French mathematician Paul Lévy, arose in a purely mathematical context in the first half of the last century^52^. They were first observed in physical systems with chaotic dynamics^53^,^54^,^55^,^56^. LW entered the biological literature when Shlesinger and Klafter^57^ proposed that they can be observed in the movement patterns of foraging ants. The physical and biological strands of LW research initiated by these studies have intersected on many occasions, most notably when attempting to identify processes underlying observations of organisms performing LW^48^. Nonetheless, chaos has so far not been a prominent element in the literature on biological LW; although Cole^23^ did report that ant activity patterns (stop-go patterns) are chaotic. This is perhaps surprising given that chaos is an ubiquitous property of neuronal systems in invertebrates such as snails and molluscs, although it remains to be seen whether or not these involve ‘motor neurons’ or are tightly linked to muscle cells^20^,^21^,^22^. Nonetheless, taken together, the insights of early disparate studies^20^,^21^,^22^,^53^,^54^,^55^,^56^ suggest that the potential for biological LW may be widespread. Here we provided support for this conjecture by showing that Weierstrassian random walks – closely linked to LW - in mud snails are associated with chaotic dynamics.

The particular coupling is not unexpected as chaos can generate Weierstrassian random walks^27^,^28^. Exponentials can be combined in many other ways so as to closely resemble LW, e.g., via Gauss-Legendre quadrature, a much-used method in numerical analysis^58^. But these approaches are not as economical as Weierstrassian random walks, requiring more exponentials to obtain accurate representations of power-laws, and more importantly appear to lack simple generative mechanisms. The occurrence and precise form of the Weierstrassian random walks will, however, depend on the nature of the chaos which in turn is sensitively dependent upon the underlying neurological/physiological processes. This plasticity suggests that Weierstrassian random walks can be tuned to optimize search efficiency, in accordance with the ‘Lévy flight foraging hypothesis’^4^,^5^. This states that because LW can optimize foraging efficiency, natural selection should have led to adaptations for Lévy flight foraging.

This notion of selection and adaptation resonates with that of Rabinovich and Abarbanel^58^ who were among the first to critique the role of chaos in neuronal systems. These authors focused their attention on the occurrence of chaos in synaptically isolated neurons in central pattern generators. They noted that chaotic neurons not only regulate each other’s behaviours, but easily adapt to extracellular parameters such as the coupling strengths among the neurons which are determined by the concentration of neuromodulators and other factors. This led Rabinovich and Abarbanel^59^ to suggest that chaos in central pattern generators is a ‘waiting state’ which arises when the connections among the chaotic neurons “turned off” while waiting for external signals with information to act on. When particular sensory stimuli are applied this state transforms into one of the many organized states (e.g., synchronized oscillations) that the neurons can support, and the chaos vanishes. When the environment changes again, other patterns emerge or the background chaos returns. Our findings suggest that molluscs have taken advantage of this chaos to produce Lévy walks when searching in the presence of minimal external stimuli. The exact neuronal generative mechanism and whether it is more or less centralized in molluscs (e.g. central pattern generator in the brain, pace-maker neurons controlling contractions in the pedal organ) needs further investigation. What is clear is that the chaotic route to Lévy walking stands apart from the many other potential routes to Lévy walking which have been identified and which seem to be rigid rather than plastic^48^.

**Figure 4:**
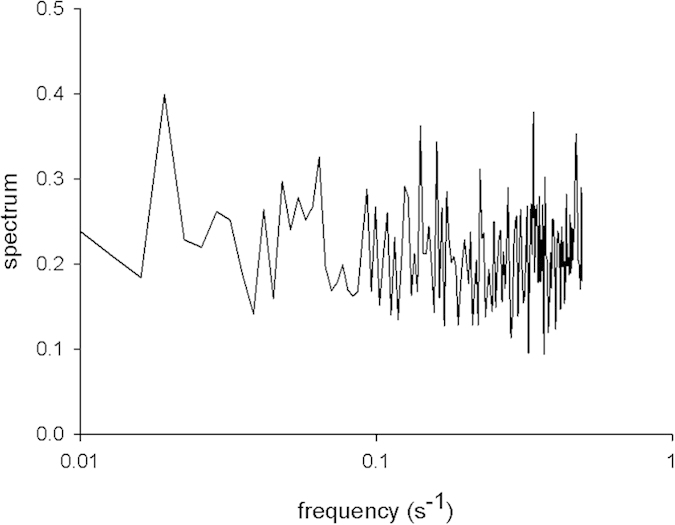
Spectrum derived from the movement pattern data collected by de Jager *et al*.^6^ for mussels during the formation of patterned beds.

For exploration of the generality of our conclusions, we additionally re-examined a dataset of 50 mussels during the process of patterned-bed formation^6^. Previously it was shown that mussel movements are well described by 3-tier Weierstrassian random walks^32^. But we could find no evidence for these movement patterns being derived from chaotic dynamics. The power-law spectrum did not have the expected, stretched exponential form but was instead flat (Fig. 4). Thus, we conclude that the hallmarks of chaos can be absent or impaired if, as in the case of mussels, the coupling between neurological processes and movement is hindered by the organisms’ physiology and/or body form constraints, i.e., for example, by being encased within a shell.

### Outlook

Despite the emergence of an extensive body of literature on the occurrence of LW movement patterns^6^,^7^,^8^,^9^,^10^,^11^,^12^,^13^,^14^,^15^,^16^ there is strong disbelieve in the validity and usefulness of the Lévy walk paradigm amongst some ecologists studying foraging behaviours^17^. Many of the classical theories of foraging are based on observations of higher animals which have extensive perceptive and cognitive abilities. A large component of their movement will be driven by observation and memory, and hence will be non-random. Random LW movement patterns have, for the most part, been observed in lower, invertebrate animals (with the exception of some marine predators that prey on species that are difficult to perceive^9^,^15^). Our study on simple invertebrate animals adds to the growing realization that LW are more likely to be observed in organisms that cannot detect their prey from any significant distance (and so need to come into more close contact with a target to establish its presence^60^) and unlike higher animals cannot use a combination of memory and landscape recognition for navigation in order to increase the likelihood of encountering food. Our results strengthen this viewpoint as chaotic neural patterns, providing a simple basis to generate seemingly complex search patterns such as a LW, are found in lower animals that have two or three paired nerve cords as their central nervous system. Under this new hypothesis, LW could provide an initial movement template, which can provide an efficient search strategy with minimal required input from observations of search targets. This warrants further investigation.

## Additional Information

**How to cite this article**: Reynolds, A. M. *et al*. Signatures of chaos in animal search patterns. *Sci. Rep*. **6**, 23492; doi: 10.1038/srep23492 (2016).
